# Wrapper-based deep feature optimization for activity recognition in the wearable sensor networks of healthcare systems

**DOI:** 10.1038/s41598-022-27192-w

**Published:** 2023-01-18

**Authors:** Karam Kumar Sahoo, Raghunath Ghosh, Saurav Mallik, Arup Roy, Pawan Kumar Singh, Zhongming Zhao

**Affiliations:** 1 Department of Computer Science and Engineering, National Institute of Technology, Mahatma Gandhi Road, A-Zone, Durgapur, West Bengal 713209 India; 2 Department of Information Technology, Jadavpur University, Jadavpur University Second Campus, Plot No. 8, Salt Lake Bypass, LB Block, Sector III, Salt Lake City, Kolkata, 700106 West Bengal India; 3 Center for Precision Health, School of Biomedical Informatics, The University of Texas Health Science Center at Houston, Houston, TX 77030 USA; 4 Harvard T.H. Chan School of Public Health, Harvard University, Boston, MA 02138 USA; 5 Department of Computer Science and Engineering, Dr. B. C. Roy Engineering College, Durgapur, West Bengal 713206 India; 6 Human Genetics Center, School of Public Health, The University of Texas Health Science Center at Houston, Houston, TX 77030 USA

**Keywords:** Health care, Engineering, Mathematics and computing

## Abstract

The Human Activity Recognition (HAR) problem leverages pattern recognition to classify physical human activities as they are captured by several sensor modalities. Remote monitoring of an individual’s activities has gained importance due to the reduction in travel and physical activities during the pandemic. Research on HAR enables one person to either remotely monitor or recognize another person’s activity via the ubiquitous mobile device or by using sensor-based Internet of Things (IoT). Our proposed work focuses on the accurate classification of daily human activities from both accelerometer and gyroscope sensor data after converting into spectrogram images. The feature extraction process follows by leveraging the pre-trained weights of two popular and efficient transfer learning convolutional neural network models. Finally, a wrapper-based feature selection method has been employed for selecting the optimal feature subset that both reduces the training time and improves the final classification performance. The proposed HAR model has been tested on the three benchmark datasets namely, HARTH, KU-HAR and HuGaDB and has achieved 88.89%, 97.97% and 93.82% respectively on these datasets. It is to be noted that the proposed HAR model achieves an improvement of about 21%, 20% and 6% in the overall classification accuracies while utilizing only 52%, 45% and 60% of the original feature set for HuGaDB, KU-HAR and HARTH datasets respectively. This proves the effectiveness of our proposed wrapper-based feature selection HAR methodology.

## Introduction

The word “Automation” has created a buzz around the world. Every industry is trying to automate its day-to-day tasks. As a result, it opens up a sizable market for invention and study on numerous subjects that aim to improve human existence through technology. To automate a process, it is important to analyze or recognize existing human activities. Human Activity Recognition (HAR) is one of the efficient ways to accomplice the challenges in this field. It is a colonist research area in computer vision that recognizes human activity through any mobile or IoT device sensors^1^.

Wireless sensor networks, a result of recent advancements in sensing technology, offer an unobtrusive, privacy-friendly, and simple to install answer to home monitoring. Contact switches are utilised as sensors to determine if doors and cabinets are open or closed, and pressure mats are used to determine whether someone is sitting or lying down^2^. Wearable sensor usage has also increased significantly recently, particularly in the medical sciences where there are many diverse applications for monitoring both psychological states and human activity. In the medical industry, it is feasible to keep an eye on patients’ vital statistics such body temperature, heart rate, brain activity, and muscle movement^3^.

**Figure 1:**
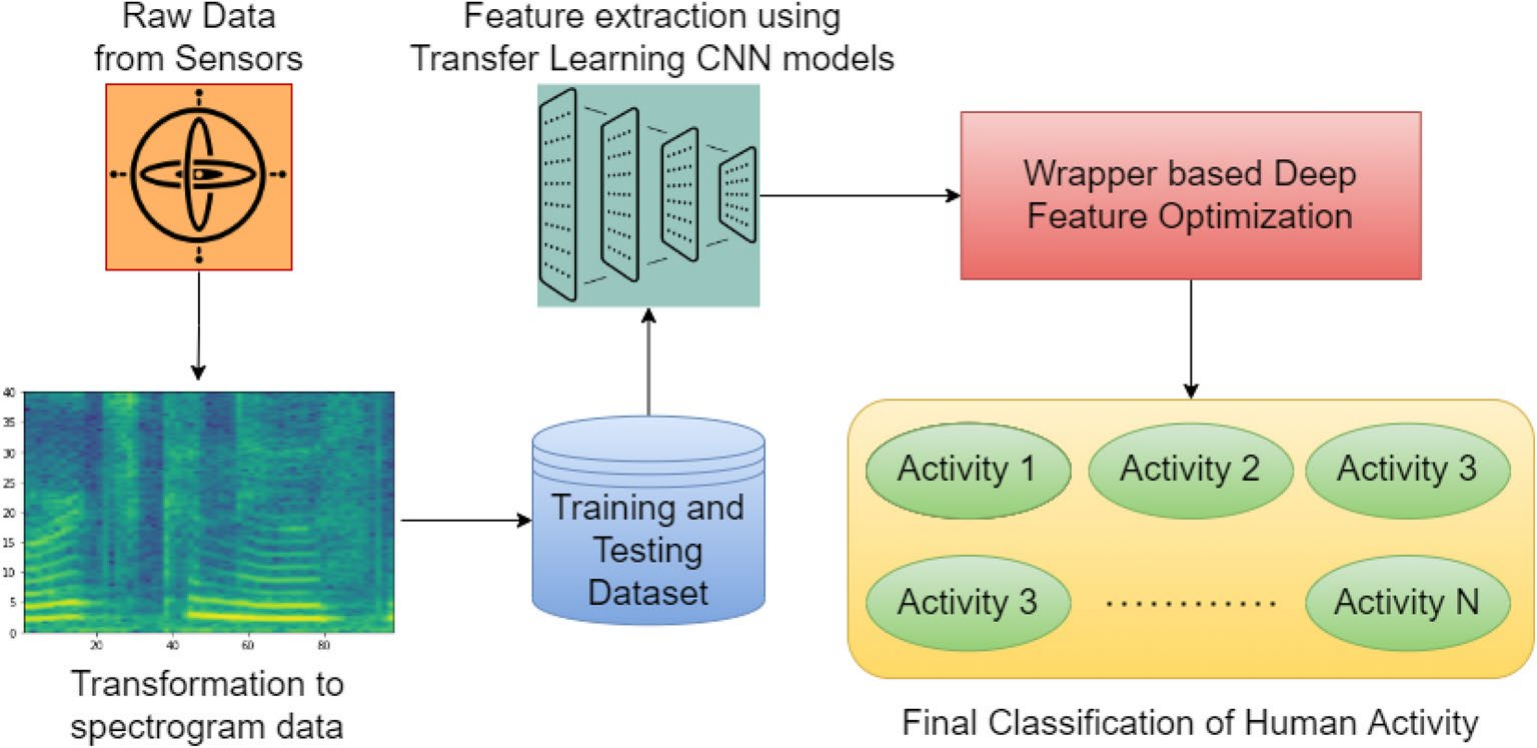
Graphical representation of the proposed wrapper-based deep feature optimization framework for HAR problem.

### Limitations of previous works

Current research on HAR is focused on deep learning and machine learning approaches because of its improved accuracy, robustness and speed compared to traditional techniques^4,5^. Several distinct deep learning approaches have emerged over the last few decades, which introduces on various parts of the HAR pipeline. Present-day state of-the-art machine learning models require an adequately large dataset for training them in order to produce accurate results. A number of diverse datasets have been published to overcome this challenge. Previous studies have investigated the problems of HAR using popular machine learning algorithms such as Random Forest (RF), Support Vector Machine (SVM), etc. and deep learning architectures such as Convolutional Neural Network (CNN), Long Short-Term Memory (LSTM), Recurrent Neural Network (RNN) and Artificial Neural Network (ANN) to name a few. Almost all machine learning algorithms face their main difficulty in the time-consuming feature engineering and human feature extraction process. The exigent need for optimized feature selection is the presence of redundant and less significant data among the complete set of available feature space. This increases the computation time for the model to train and the presence of noise reduces the performance. However, feature selection is not synonymous with dimensionality reduction since the former does not alter the attributes of the feature space but just removes them to reduce model complexity. Various research fields have seen the application of feature selection to solve numerous problems such as COVID-19 detection^6^, Indian spoken language identification^7,8^ and speech emotion recognition^9^. The robustness and accuracy that feature selection provides to select deep learning features is leveraged to identify human activities, which has seen little application of feature selection. The main advantages of feature selection are:

- Reduces the possibility for the model to train noise and redundant features.
- Improves accuracy of the model by eliminating less important data.
- The machine learning or deep learning models can train faster since they can train fewer number of datasets.

In the context of supervised learning, feature selection can be divided into the following broad categories:

- Filter method: The link between each input variable and the target variable is assessed using statistical techniques using filter feature selection methods, and the scores obtained are then used to choose (filter) the input variables that will be incorporated in the model.
- Wrapper method: In the wrapper technique, features are chosen by treating them as a search problem, where many combinations are created, assessed, and contrasted with other combinations. Iteratively employing the subset of characteristics trains the algorithm. Recursive feature removal and genetic algorithm are a few examples.
- Embedded Method: By taking into account feature interaction and low computing cost, embedded techniques incorporated the benefits of both filter and wrapper approaches. These are quick processing techniques that are comparable to the filter approach but are more precise.

The goal of detecting various human activities is better suited to deep learning techniques because they automatically train the features from the images or data. In this paper, two popular transfer learning models have been chosen for feature extraction due to the robustness and advancement of deep learning. Transfer learning is a type of supervised learning, where the input remains the same but the target output can be of some other nature.

As transfer learning model frameworks can yield a certain set of features, some of which may be identified to be redundant^10^ using Binary Bat Algorithm (BBA). We have proposed a hybrid model using a combination of transfer learning models^11^ and a wrapper optimizer algorithm based on BBA, to build an efficient model in order to identify human activities. The schematic diagram of our proposed work is also shown in Fig. 1. The entire framework can be further divided into three sub-modules.

1. Conversion of both accelerometer and gyroscope sensor data into spectrogram^12^ images.
2. Extraction of the deep features using two pre-trained transfer learning models.
3. Selection of optimal features using wrapper-based optimization method using BBA^13^ and classification of each activity by the optimal feature subset.

We have experimented on three standard and recently developed HAR datasets as benchmark, which are: Human Activity Recognition Trondheim (HARTH)^14^, Khulna University-HAR (KU-HAR)^15^ and Human Gait DataBase (HuGaDB)^16^ using the proposed framework and the same has been mentioned in later sections of this paper.

This paper is structurally arranged as follows: A brief literature review to highlight prior efforts on sensor-based data for the HAR problem is presented in Literature Analysis, whereas the architectural details of our proposed model along with the dataset description are presented in Materials and Methods. Experimental Results Section provides the experimental findings attained by the proposed HAR framework. Finally, the comprehensive summary of our work and future research are concluded in Conclusions & future works.

### Motivation of proposed work

Researchers used several machine learning and CNN-based models in their work, as was discovered after examining the prior research on these datasets. For all three HAR datasets, no generic model was applied. The sensor-based HAR problem has been transformed into an image classification problem using a robust model that first transforms the sensor data into the corresponding spectrogram images. As per our knowledge, this type of transformation (from raw sensor data to spectrogram images) has been done for the first time for solving HAR problem. Following the above transformation, we suggest a two-fold model in which

- For feature extraction from the spectrogram images, Efficientnet_b0 and Mobilenet_v3_Large, two well-known transfer learning-based models, are applied.
- The best optimal feature subset from the retrieved feature set is chosen using the BBA. Finally, the classification of human activities has been carried out using the optimal feature subset.

The contributions of the proposed research are as follows:

1. The proposed work transforms the raw sensor data into its corresponding spectrogram images, which is done for the first time in the domain of sensor-based HAR.
2. The compact input images are fed as input to both Efficientnet_b0 and Mobilenet_v3_Large architectures for deep feature extraction from the penultimate layer of the networks since, due to the less amount of available data, an end-to-end classification scheme using CNN models fail.
3. The extracted features from the two pre-trained transfer learning models (trained with two different compact inputs) have been concatenated to form a final feature space, which is then fed into the BBA for the selection of an optimal feature subset.
4. The final classification on the optimal feature set is done by using K-nearest neighbor (KNN) classifier, thus achieving commendable results on the three publicly available HAR datasets that have been used to evaluate the proposed sensor-based HAR model.

## Literature analysis

HAR is one the important and highly researched field of computer vision since its beginning. The aim of HAR system is to identify activities performed by a person. HAR finds application in various areas like health monitoring, gesture based systems, intuitive interfaces for machines (gaming consoles), and surveillance-based security. It has evolved to the point where the recognized method can be generalized and has accurate recognition of human activity as possible^17^.

The HAR methodology’s complexity is related to the various data inputs that can come from different modalities such as videos, images, audio signals, wearable sensors and other sources. Based on various experiments performed by researchers, many HAR models have been developed in order to improve the overall performance and quality metrics^18^. Several distinct deep learning approaches have emerged over the last few decades, each of which innovates on different areas of the HAR pipeline^19^. Generally, deep learning methodologies emphasises on features whereas Reinforcement learning emphasises on feedback. On the other hand, traditional machine learning focuses on reaping the benefits of planting fruits and beans, whereas transfer learning can draw inferences from both. Recently, deep learning models are being used in various research areas of computer vision as well as pattern recognition domains. For solving HAR problem, Mukherjee et al.^20^ have developed an ensemble model named as EnsemConvNet in their work and achieved recognition accuracy of about 97% on WISDM dataset. Furthermore, Das et al.^21^ proposes a multi-modal HAR model called MMHAR-EnsemNet and able to achieve accuracy of about 99% on both UTD-MHAD and Berkeley-MHAD datasets. Bhattacharya el al.^22^ proposed a deep learning model, named as SV-NET, in order to recognize the human activities from video images. Banerjee et al.^23^ proposed a fuzzy integral-based CNN classifier model for skeleton-based HAR problem. Additionally, different applications of CNN models for solving image classification problems proposed by Bhattacharya et al.^24^, Chattopadhyay et al. ^25,26^. Channel equalization and channel selectivity in CNNs have been employed for the first time in HAR domain by Huang et al.^27,28^ in order to reactivate the channels that collapse due to normalization. Huang et al.^29^ also optimized the CNN feature layers using feature activation to boost accuracy of their HAR model by inhibiting filters that contribute less towards classification performance^6^, Mondal et al.^30^ and Chakraborty et al.^31^ in their respective work. Looking at wide applications of deep learning models, a hybrid model is proposed which is a combination of deep learning and wrapper-based optimization method for solving HAR problem from wearable sensor data. In the proposed model, two standard pre-trained transfer learning models such as Mobilenet_v3^32^ and Efficientnet_b0^33^ are being used for feature extraction and a BBA is used to optimize the original feature set. During the experiment, three publicly available HAR datasets are used to train and validate the model. The three HAR datasets, used in the present work, are as follows:

1. HARTH[9] dataset
2. KU-HAR[10] dataset
3. HuGaDB[11] dataset

HARTH dataset was recently introduced by Aleksej Logacjov et al.^14^ in November 2021. The primary goal of their research is to present a new accelerometer-based publicly available HAR dataset which can be considered as a free-living dataset because accelerometer-based HAR datasets are very less according to Stricker and Micucci et al.^34^. The recent survey confirmed this, revealing that only 30 out of 142 accelerometer-based datasets were publicly available^35^. The authors trained the dataset by using state-of-the-art machine learning models and were able to achieve the best F1-score of 81% by using a SVM classifier. Like the HARTH dataset, we have selected the KU-HAR dataset which got recently developed in March 2021 by Niloy Sikder et al.^15^. Their main aim was to introduce a new smartphone sensor data (based on both gyroscope and accelerometer) with new activity classes that will assist researchers in developing more delicate models for designing a real-world HAR framework. They achieved nearly 90% accuracy by using RF classifier.

Apart from the HARTH and KU-HAR datasets to test the robust behaviour of our proposed model, we have selected the standard HuGaDB dataset which was introduced by Roman Chereshnev et al.^16^ in July 2017. This is a human gait data collection made freely available in UCI Machine Learning repository. Gochoo et al.^36^ applied hierarchical feature-based technique to extract the feature which is then optimized using Stochastic gradient descent (SGD) technique and attained 92.50% accuracy. A hybrid feature selection model using deep belief networks was proposed by Madiha Javeed et al.^37^ and able to achieve 92.5% accuracy on HuGaDB dataset. Bin Fang el al.^38^ applied CNN-based model and achieved 79.24% accuracy. Yingnan Sun et al.^39^ proposed a novel ANN based classification model for real-time gait analysis and achieved 88% accuracy. Girja Kumari et al.^40^ used LSTM based deep learning classifier model in their proposed work and achieved 91.1% accuracy on HuGaDB dataset. Several researchers used the same dataset for their research. A brief summarization of existing HAR models, developed in the literature, for HARTH, KU-HAR and HuGaDB datasets are included in Table 1.

**Table 1:** A tabular summary of different HAR methodologies and their corresponding performances (in terms of accuracy) achieved till date for HARTH, KU-HAR and HuGaDB datasets.

Dataset	Study	Method	Accuracy(%)
HARTH	Logacjov et al.^14^	SVM-based classification model	81
KU-HAR	Sikder and Nahid^15^	RF-based classification model	89.67
HuGaDB	Filtjens et al.^41^	Multi-stage spatial-temporal graph convolutional network (MS-GCN)	83.8
	Gochoo et al.^36^	Hierarchical feature-based technique with SGD	92.5
	Javeed et al.^37^	A Hybrid features selection model using deep belief networks	92.5
	Fang et al.^38^	CNN model	79.24
	Sun et al.^39^	ANN based method for real-time gait analysis	88
	Kumari et al.^40^	LSTM model	91.1

## Materials and methods

### Dataset description

In our proposed work, we have experimented with three publicly available HAR datasets:

1. HARTH^14^ dataset
2. KU-HAR^15^ dataset
3. HuGaDB^16^ dataset

The detailed discussion related to the above-mentioned HAR datasets is described below.

#### HARTH dataset

**Figure 2:**
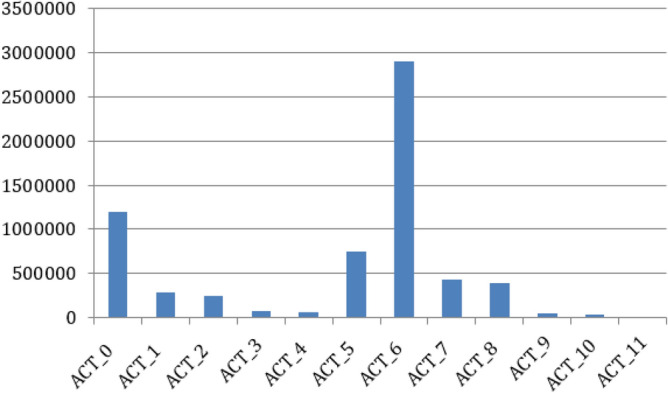
Class-wise distribution of human activities in the HARTH dataset.

The HARTH dataset is accessible to the general public. A total of 22 individuals who wore two three-accelerometers on their lower back, and thigh provided the acceleration data for this report. The HARTH dataset has 12 human activities or classification labels. Activities and their corresponding IDs are described in Table 2 whereas the number of samples per activity class present in HARTH dataset are shown in Fig. 2.

**Table 2:** A tabular representation of the classes of human activities performed in the HARTH dataset.

Activity ID	Activity
ACT_0	Walking
ACT_1	Running
ACT_2	Shuffling
ACT_3	Stairs (ascending)
ACT_4	Stairs (descending)
ACT_5	Standing
ACT_6	Sitting
ACT_7	Lying
ACT_8	Cycling -Sit
ACT_9	Cycling-Stand
ACT_10	Cycling-Sit-Inactive
ACT_11	Cycling-Stand-Inactive

#### KU-HAR

**Figure 3:**
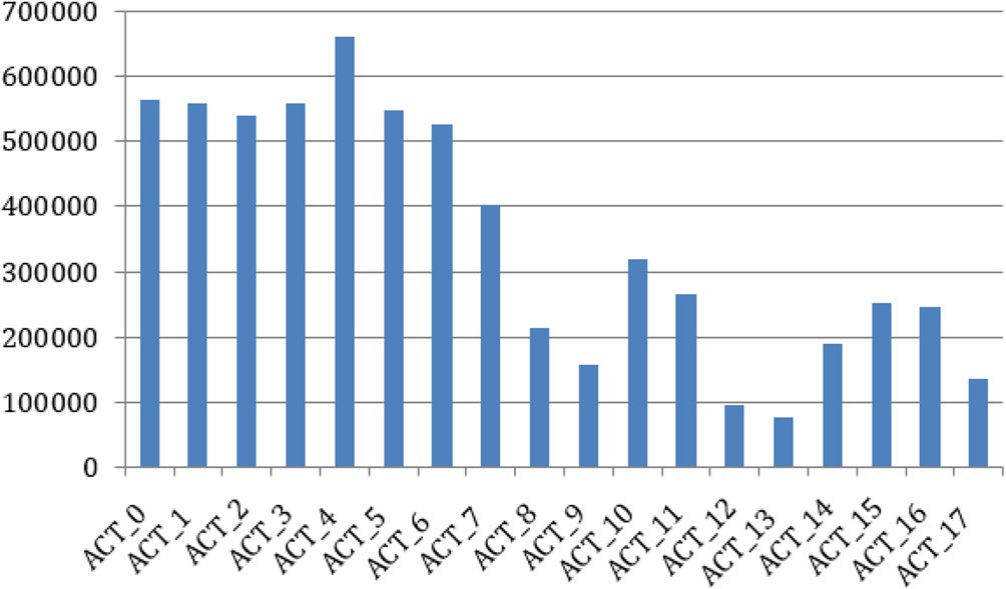
Class-wise distribution of human activities in the HARTH dataset.

A set of 90 participants (involving 75 men and 15 women) submitted data on 18 different activities using the smartphone sensors, such as the accelerometer and gyroscope. Activities and their corresponding IDs are described in Table 3 whereas the number of samples per activity class present in KU-HAR dataset are shown in Fig. 3.

**Table 3:** A tabular representation of the classes of human activities performed in the KU-HAR dataset.

Activity ID	Activity
ACT_0	Stand
ACT_1	Sit
ACT_2	Talk sit
ACT_3	Talk stand
ACT_4	Stand sit
ACT_5	Lay
ACT_6	Lay Stand
ACT_7	Pick
ACT_8	Jump
ACT_9	Push-Up
ACT_10	Sit-Up
ACT_11	Walk
ACT_12	Walk-Backward
ACT_13	Walk-Circle
ACT_14	Run
ACT_15	Stair-Up
ACT_16	Stair-Down
ACT_17	Table-Tennis

#### HuGaDB Dataset

**Figure 4:**
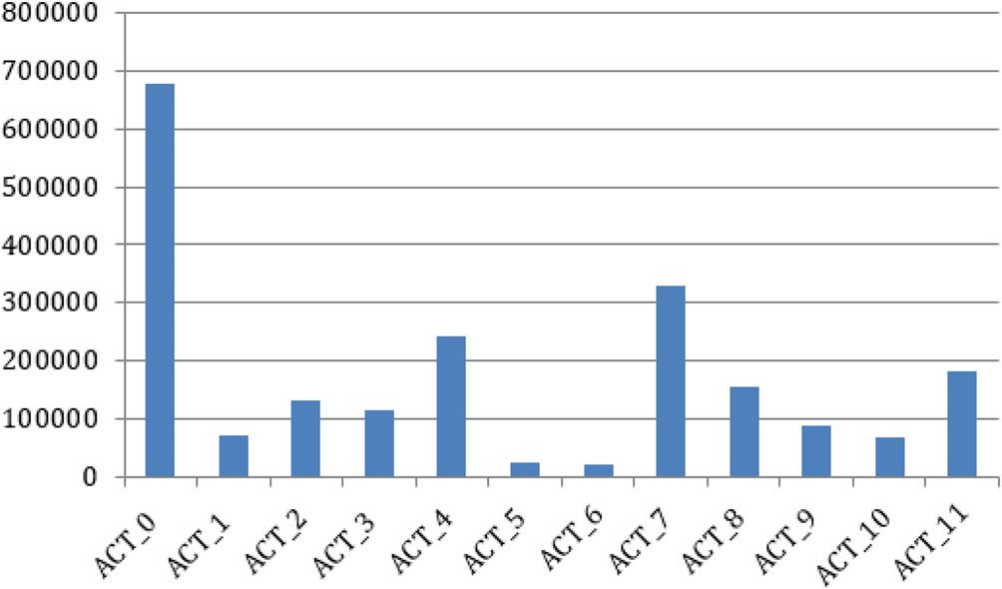
Class-wise distribution of human activities in the HuGaDB dataset.

This dataset includes continual recordings of a variety of activities, including standing up, walking, and utilizing the stairs, etc.. The data was gathered using a six-wearable body sensor system, which included inertial sensors placed on the thighs, feet, and right and left shins. Two EMG sensors were also attached to the quadriceps to track muscle activation as well. Activities and their corresponding IDs are described in Table 4 whereas the number of samples per activity class present in HuGaDB dataset are shown in Fig. 4.

**Table 4:** A tabular representation of the classes of human activities performed in the HuGaDB dataset.

Activity ID	Activity
ACT_0	Walking
ACT_1	Running
ACT_2	Going-up
ACT_3	Going down
ACT_4	Sitting
ACT_5	Sitting down
ACT_6	Standing-up
ACT_7	Standing
ACT_8	Bicycling
ACT_9	Up by elevator
ACT_10	Down by elevator
ACT_11	Sitting in car

### Proposed model

**Figure 5:**
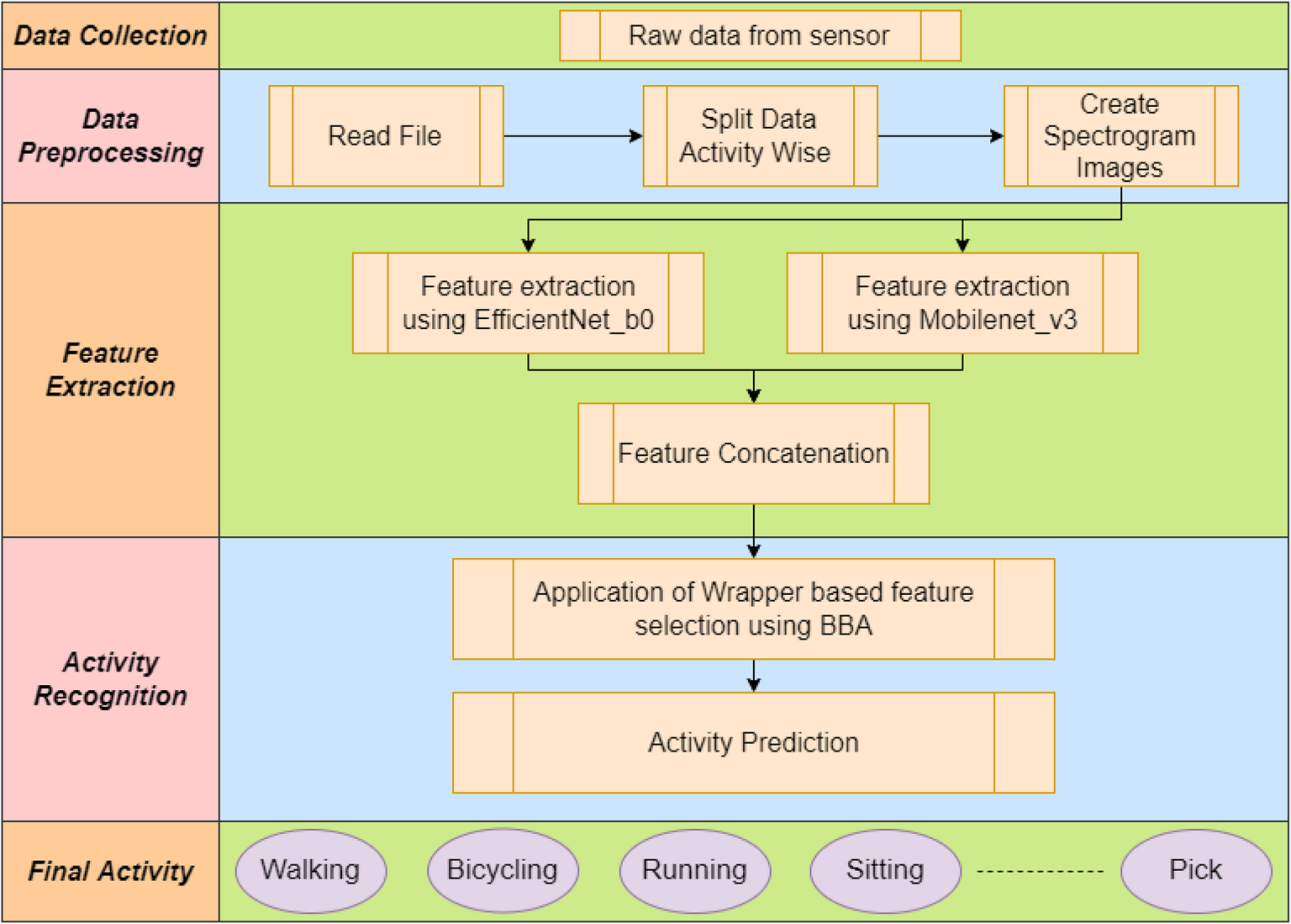
A graphical visualization of the entire proposed wrapper-based deep feature optimization framework for HAR.

A diagrammatic illustration of the entire proposed HAR framework, from data acquisition, transfer learning^11^ to final classification using wrapper-based deep feature selection algorithm has been given in Fig. 5.

The entire framework can be subdivided into three phases:

1. **Data Pre-processing:** Transform the raw sensor time-series data into spectrogram images.
2. **Feature Extraction:** Extract deep features using two popular transfer learning model (Mobilenet_v3_Large and Efficientnet_b0) followed by concatenation of extracted features.
3. **Feature Selection:** Application of wrapper-based deep feature selection using BBA and classification of the target activities performed.

#### Data pre-processing

**Figure 6:**
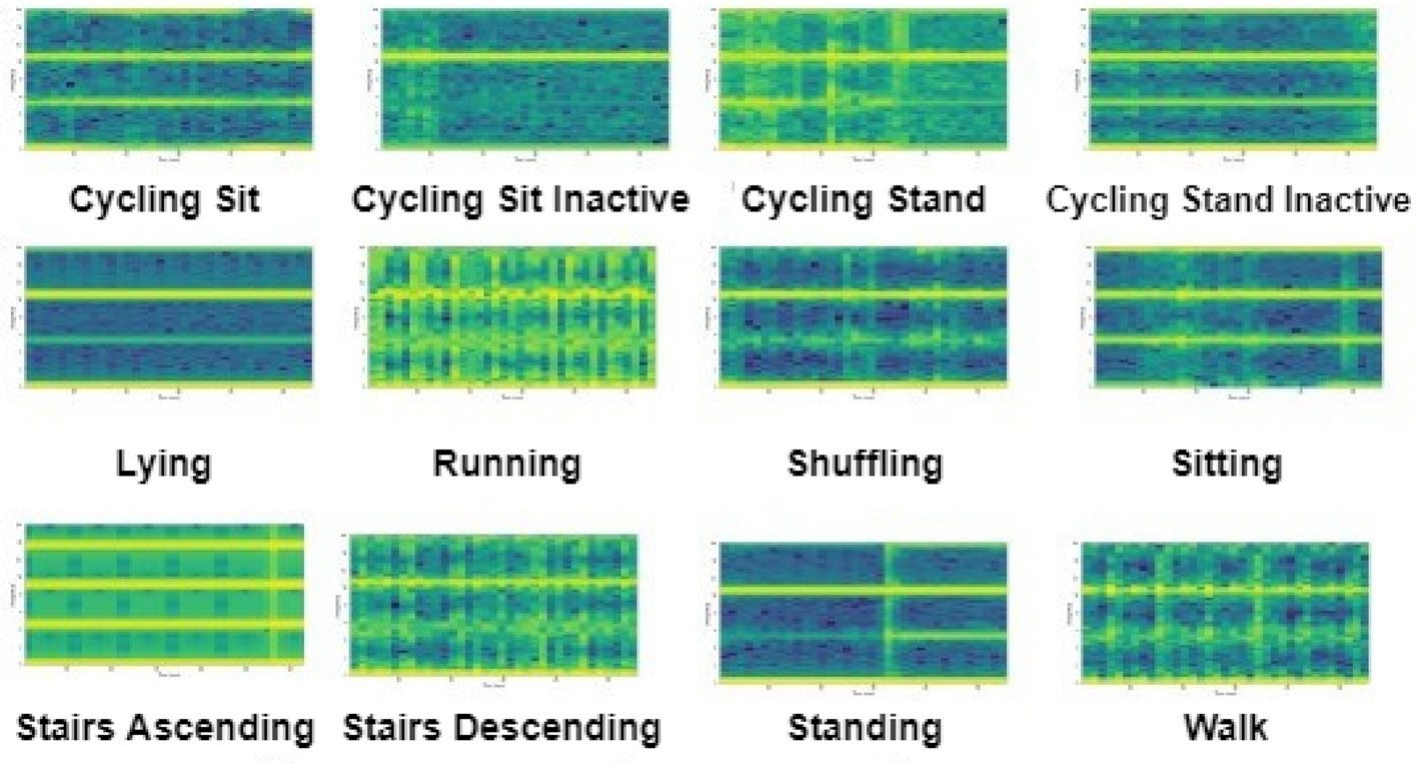
Spectrogram images for various human activities from the HARTH dataset.

**Figure 7:**
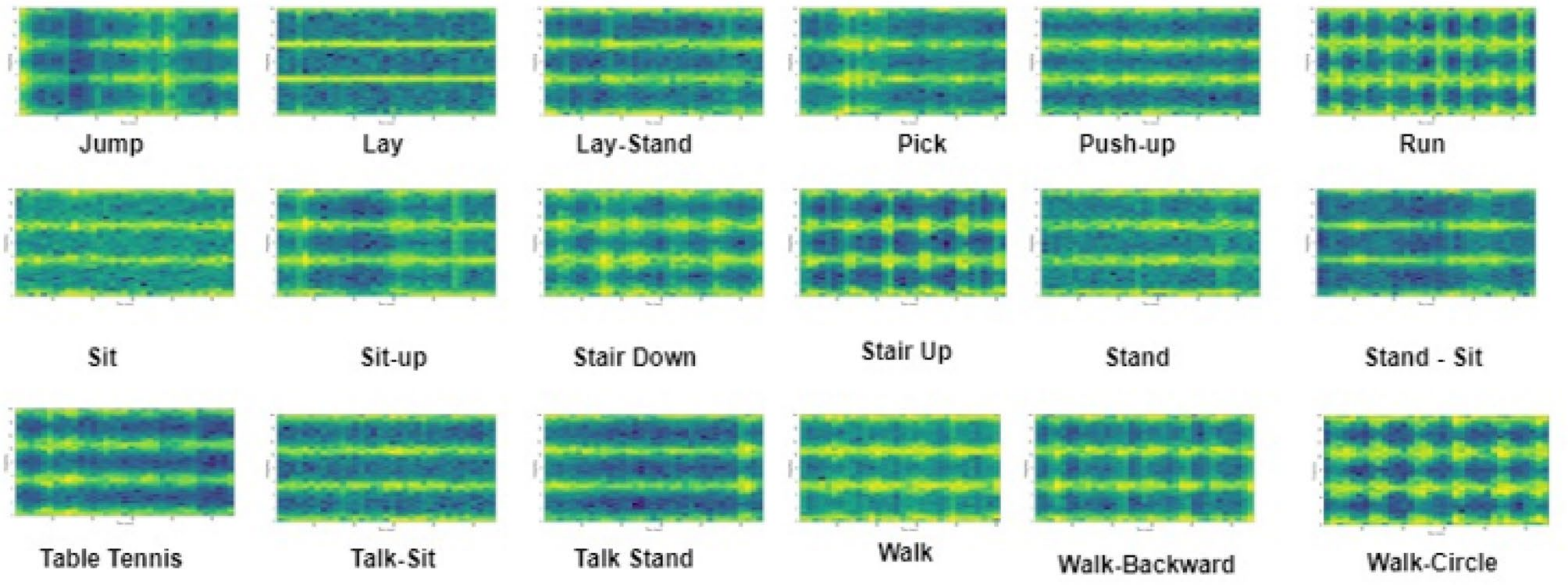
Spectrogram images for various human activities from the KU-HAR dataset.

**Figure 8:**
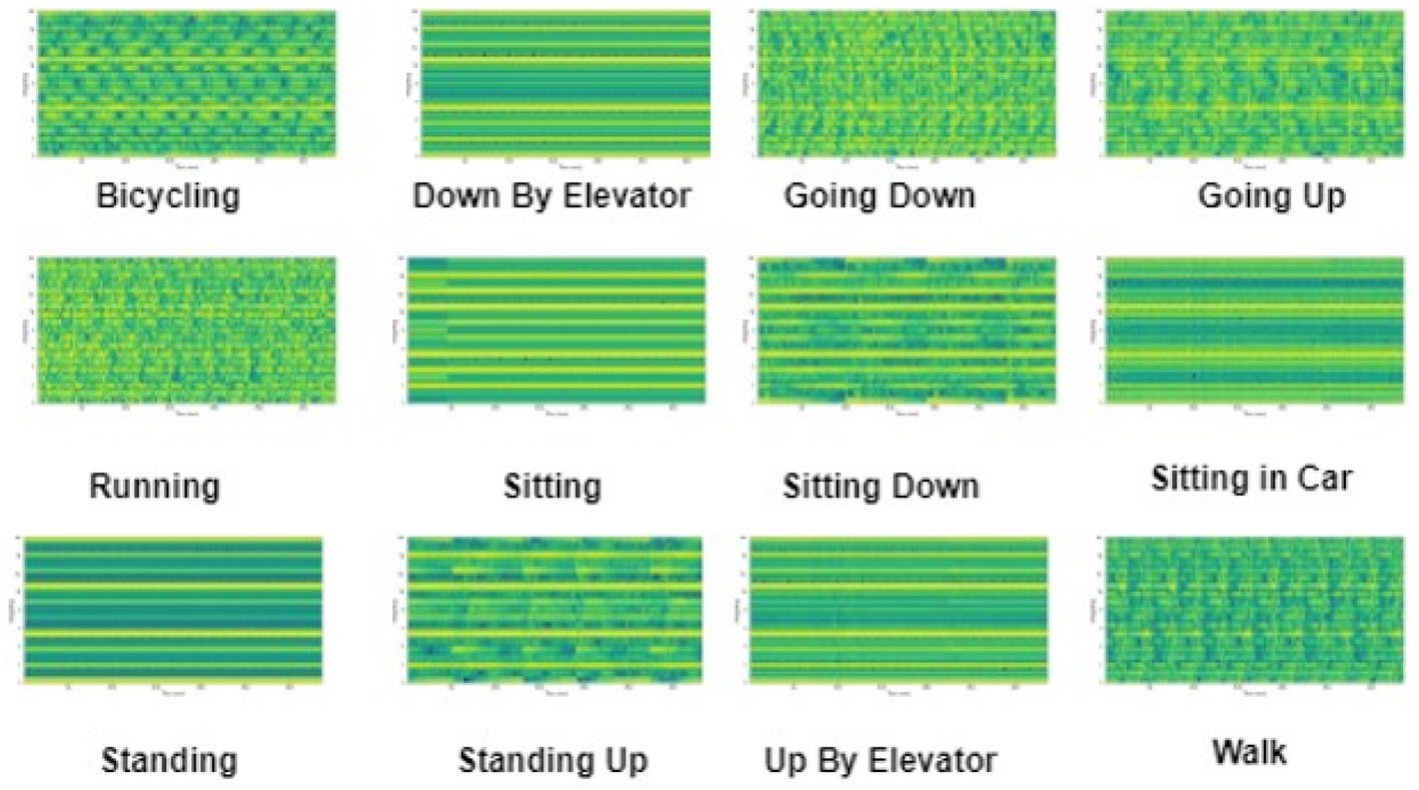
Spectrogram images for various human activities from the HuGaDB dataset.

We employ two widely used transfer learning models that need images for training; as a consequence, raw sensor data are transformed into spectrogram images. As mentioned earlier, three recently developed HAR datasets such as HARTH, KU-HAR, and HuGaDB are being used in this work. The two sensor values (acceleration and gyroscopic) are present in all three HAR datasets. First, all sensor readings have been gathered activity-wise in the various data arrays using the raw data that has been received from the file. Both the generation of spectrogram images as well as activity-wise data arrays need the division of data arrays into numerous data frames (300 rows, in our case). The dataset’s smallest activity data length is calculated among the activities that are regarded to have the most rows overall during this splitting. The prior step is crucial for ensuring a balanced distribution of samples among each activity class. The purpose of this step is to encode a spectrogram from time series data using the Pylab^42^ Python package. Figures 6, 7 and 8 show samples of spectrograms generated for each human activities from the HARTH, KU-HAR and HuGaDB datasets respectively.

#### Feature extraction

Spectrogram images generated in the previous phase are fed to the Mobilenet_v3_Large and Efficientnet_b0 CNN transfer learning models. Following this, the extracted features are saved for each model separately for building the further phases of the total framework. Transfer learning models are trained on millions of image data samples as a result of which they can classify image or similar data with high performance. Hence, the sensor data modalities are converted to spectrogram images to leverage the pre-trained weights of the transfer learning CNN models.

##### Efficientnet

**Figure 9:**
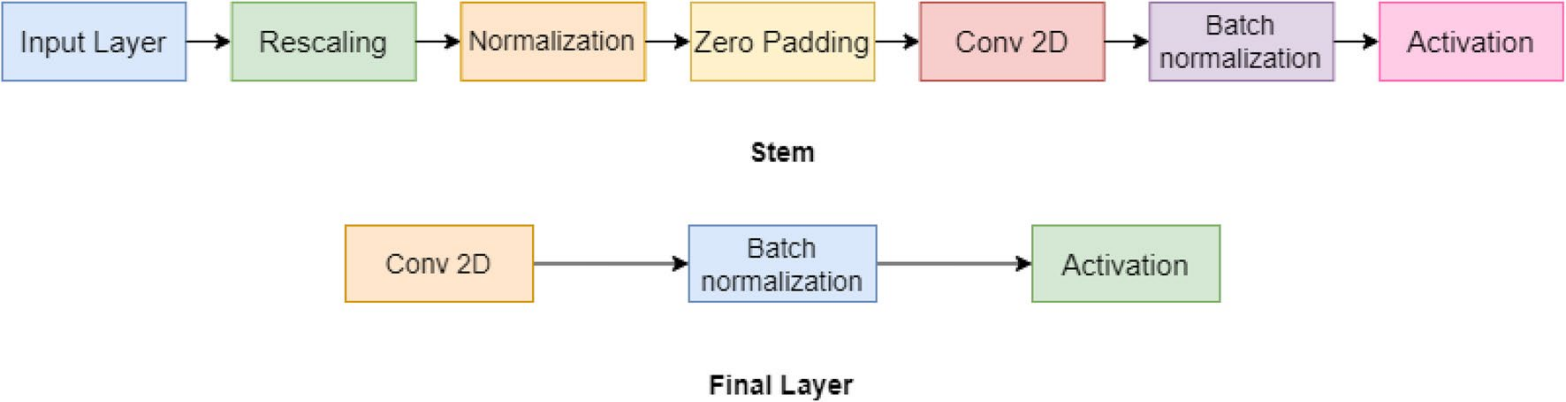
Block diagram illustration of the Efficientnet model used in the proposed HAR framework.

The new model, termed Efficientnet, has been introduced by Google^33^ with the primary goal of introducing one that is more effective than the current state-of-the-art results. Usually, the models are made with an excessively high resolution, or they are overly deep and wide. The Google study describes a scaling strategy called compound scaling, which claims that intentionally scaling all three model attributes-depth, breadth, and resolution-at the same time produces better results than scaling any one of them alone. The fused-MB convolutional layers are typical of the EfficientnetV2, which can employ current GPU/CPU accelerators and has fewer parameters and FLOPS. The original Efficientnet model has eight different blocks from b0 to b7 as shown in Fig. 9. Any network’s stem is its primary component. From there, all experiments start with the architecture of the network, which is a trait shared by all eight models, and then with the network’s final layers. Each of them has seven blocks after that. To train our spectrograms, we use the Efficientnet_b0 base model, which comprises seven inverted residual blocks, each with a different configuration. These blocks also incorporate excitation, squeeze, and swish activation. Each of them comprises seven blocks after that. In addition, each of these blocks has a different number of sub-blocks, whose total number rises from Efficientnet_b0 to Efficientnet_b7.

##### Mobilenet_v3

Mobilenet^43^ is a type of convolutional neural network which is built for embedded vision systems and mobile applications. It is based on a streamlined architecture that employs depth-wise separable convolutions to construct lightweight deep neural networks with low latency for embedded and mobile devices. To cut down on the number of parameters, a depth-wise convolution has been implemented^44^. The procedure has been equally divided into two parts^45^,

1. The depth-wise convolution
2. The point-wise convolution

**Figure 10:**
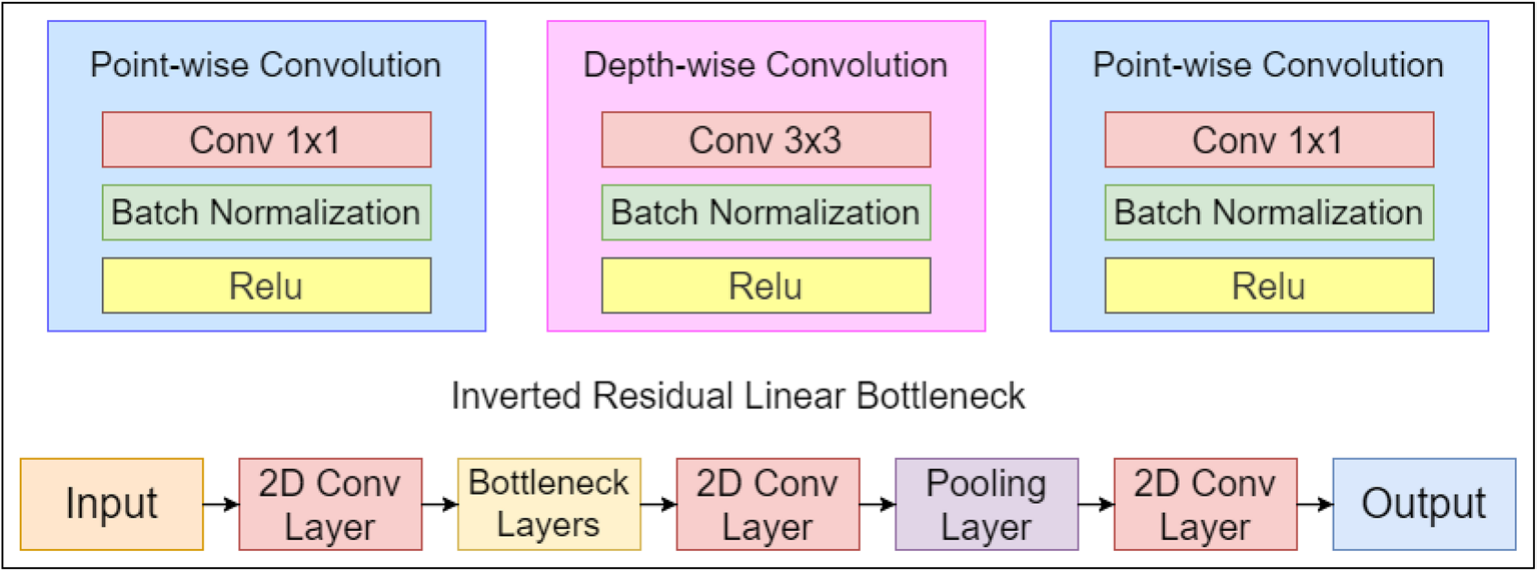
Architectural representation of the Mobilenet_v3 CNN model employed in the proposed work.

Every channel’s image input of shape (256 $\times$ 256) is filtered by depth-wise convolution then the point-wise convolution is put in a 1 $\times$ 1 convolution to integrate the output results of the previous layer. This results in a significant reduction in both model complexity as well as computational power. To improve the Mobilenet architecture, a upgraded version was introduced in 2019 named as Mobilenet_v3. and it was built by removing complex layers with employing the H-swish function rather than standard ReLU. Figure 10 summarizes the architecture of Mobilenet_v3 model used in the present work.

Mobilenet_v3 is defined as two models: Mobilenet_v3_Large and Mobilenet_v3_Small. In terms of both performance and accuracy, Mobilenet_v3_Large model gives better result. In the present work, we have selected Mobilenet_v3_Large model to train our model.

#### Selection of optimal features using BBA

In our proposed paper we have utilized the wrapper function over a filter based methodology for the selection of features due to the following reasons:

- The filter based methods use statistical methods hence there is no interaction with the model for the optimal selection of feature subsets. Contrary to this, the wrapper methodologies interact with the models by comparing every conceivable feature combination to the evaluation criterion by employing a greedy search methodology.
- While filter selection takes into account each feature separately, wrapper-based approaches take into account the dependencies between the features throughout the entire feature space, which results in improved computational performance.

Following the extraction of both model features of tensor size 1000, which is equal to the final linear layer of the two transfer learninig moedels, a single feature vector is created of tensor size 2000, which is then fed to the BBA^46^ wrapper function to select the best optimal feature subset followed by the final classification of human activities using a k-nearest neighbours classifier. The KNN algorithm^47^ is a supervised learning classifier that employs proximity to produce classifications or predictions about the grouping of a single feature point. Although it can be applied to classification or regression issues, it is commonly employed as a classification algorithm because it relies on the idea that comparable points can be discovered close to one another. The details of our experimental outcomes are included in the Results section.

##### Binary Bat Algorithm (BBA)

**Figure 11:**
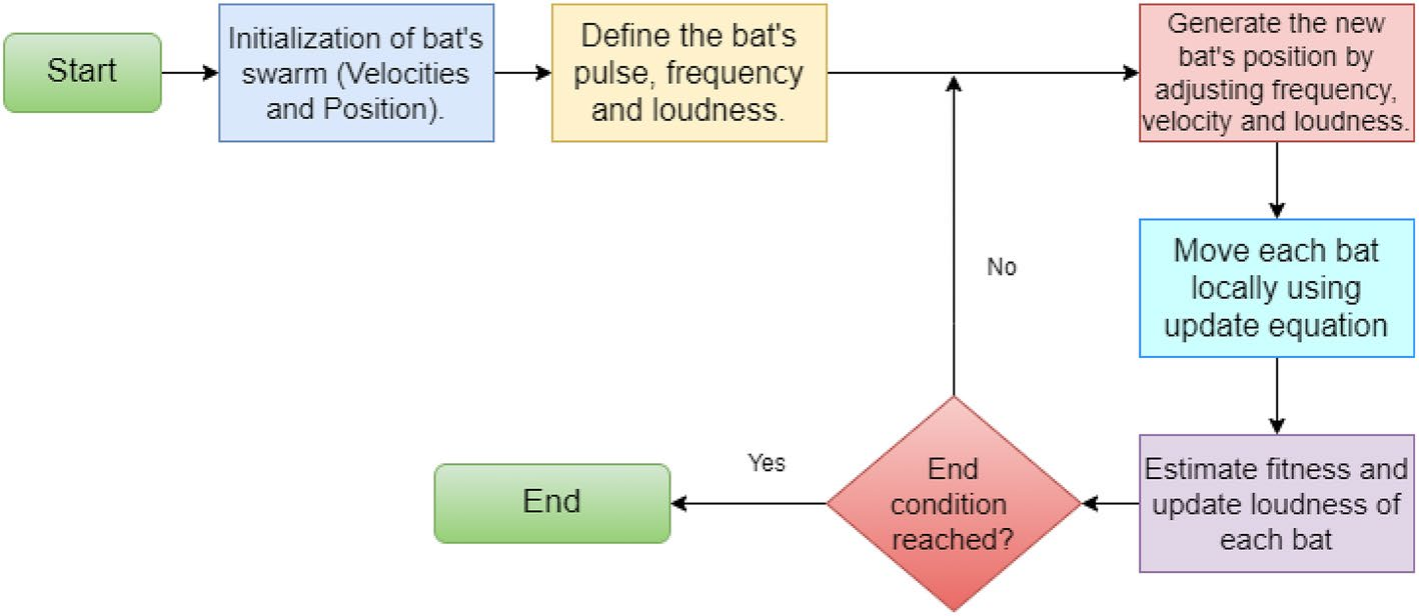
Flowchart representation of the meta-heuristic BBA used for feature selection in our proposed work.

Yang et al.’s^48^ Bat Algorithm (BA), a novel meta-heuristic approach for continuous optimization, was based on the remarkable ability of microbats to detect their food and differentiate between various bug species-even in total darkness. Such an approach has shown to be more successful than several well-known optimization techniques drawn from nature. The basic BA flowchart is shown in Fig. 11. As per BA,

1. Each bat randomly assigned a frequency between [$fe_{\text {min}}$, $fe_{\text {max}}$].
2. Every bat is associated with velocity ($V_{j}^{t}$) and position $P_{j}^{t}$ in search space at each iteration t , with respect to frequency $fe_{j}$.
3. Hence, at each iteration, we need to update $fe_{j}$ , $V_{j}$ and $P_{j}$ as per following equations.

1 $$\begin{aligned} fe_{j} = fe_{\text {min}} + \left( fe_{\text {max}} - fe_{\text {min}}\right) \beta \end{aligned}$$

2 $$\begin{aligned} V_{j}^{t} = V_{j}^{t-1} + \left( P_{j}^{t-1} - P *\right) fe_{j} \end{aligned}$$

3 $$\begin{aligned} P_{j}^{t} = V_{j}^{t} + P_{j}^{t-1} \end{aligned}$$

where, $\beta$ is random value which lies between [0, 1] and P$*$ is current best.

Later, BBA was introduced by Nakamura et al.^48^. They changed the equation position of the basic BA and replaced with binary vectors by using one of the transfer functions, and otherwise it is structurally similar to the basic BA. The optimal result has been selected among the 2*n* possibilities.

4 $$\begin{aligned} TF(V_{j}^{t}) = \frac{1}{1 + e^{-V_{j}^{t}}} \end{aligned}$$

Therefore, Eq. (3) of BBA can be replaced with Eq. (4), which is as follows:

5 $$\begin{aligned} P_{j}^{t} = \left\{ \begin{array}{c} 1 \, if\, TF(V_{j}^{t}) > \alpha \\ 0 \, if\, otherwise \end{array}\right. \end{aligned}$$

In Eq. (5), the value ’1’ indicates the feature is selected whereas ’0’ indicates that the feature is not selected, and $\alpha$ is Uniform (0, 1).

In the present work, we have chosen meta-heuristic BBA for the purpose of deep feature optimization due to the following reasons:

1. It is accurate and very efficient algorithm to solve complex problems.
2. Efficient to solve multi-stage, multi-machine , multi-product scheduling problems.
3. The nature of automatic zooming effective parameter control, the frequency tuning and echolocation are grate thins to solve wide range of problems with quick time in promising optimal solution.

### Ethics approval and consent to participate

All experiments and methods were carried out in accordance with relevant guidelines and regulations.

## Results

### Evaluation metrices

The performance of our proposed wrapper-based deep feature optimization HAR framework has been evaluated using four well-known evaluation metrics Accuracy^49^, Precision^49^, Recall^49^, and F1-score^49^. Training and validation loss are also one of the most used statistic for evaluating the efficiency of any deep neural networks; as a result, it is also employed in our proposed work. The next subsections compare our proposed model to previous preceding frameworks and architectures in-depth.

To create the aforementioned assessment metrics, simple parameters like True Positive (TP), True Negative (TN), False Positive (FP), and False Negative (FN) can be employed.

The model’s performance is shown in accuracy for all activity classes. The ratio of the number of accurate predictions to all testing samples is calculated.

6 $$\begin{aligned} Accuracy = \frac{TP + TN}{TP + TN + FP+FN}\times 100 \end{aligned}$$

Precision is also called as positive predictive value. It depicts the model’s accuracy in classifying a sample as positive.

7 $$\begin{aligned} Precision = \frac{TP}{TP+FP} \end{aligned}$$

Sensitivity is also known as recall. The recall shows how well the model can identify positive samples.

8 $$\begin{aligned} Recall = \frac{TP}{TP+FN} \end{aligned}$$

A model’s recall and accuracy are combined by the F1-score, which also calculates the harmonic mean of the model’s recall and precision.

9 $$\begin{aligned} F1-score=\frac{2}{\left( \frac{1}{Recall} \right) + \left( \frac{1}{Precision} \right) } \end{aligned}$$

### Experimental results

Tables 5 and 6 provide the results of the experiment attained by the proposed wrapper-based deep feature optimization HAR framework. Table 5 shows how the number of features decreases with the use of the BBA, which chooses the most pertinent features from all those obtained from both the Mobilenet_v3 Large and Efficientnet_b0 models. If we examine the reduction of concatenated features by dataset, it can be found that almost 52% of HuGaDB dataset, 45% of KU-HAR dataset, and 60% of HARTH dataset has been optimally selected by the BBA. Furthermore, the overall classification accuracy of the proposed model has also been improved in case of all the three HAR datasets. Table 6 illustrates the increment in the overall classification accuracies achieved by the proposed wrapper-based deep feature optimization framework for all the HAR datasets. It can be observed from Table 6 that increments of about 20%, 21% and 6% in the overall classification accuracies have been noted for HARTH, KU-HAR and HuGaDB datasets respectively which is quite impressive. The proposed HAR model has also been assessed using other common performance measures such as F1-score, Recall, and Precision, which are presented in Table 7. It can be noticed from Table 7 that the application of wrapper-based feature selection using BBA on the concatenated feature vector produces Precision, Recall and F1-score of 0.90, 0.89 and 0.89 respectively for HARTH dataset, 0.97, 0.96 and 0.96 respectively for KU-HAR dataset, and 0.94, 0.93 and 0.93 respectively for HuGaDB dataset.

**Table 5:** Epoch size along with the number of features selected before and after applying BBA based feature selection method for all the three HAR datasets.

Dataset	Model	#Runs with epoch	#Features before using BBA	#Features after using BBA
HARTH	Mobilenet_v3_large	40	960	471
	Efficientnet_b0	40	1280	464
	Concatenated feature vector	40	2240	906
KU-HAR	Mobilenet_v3_large	40	960	499
	Efficientnet_b0	40	1280	721
	Concatenated feature vector	40	2240	1249
HuGaDB	Mobilenet_v3_large	40	1280	621
	Efficientnet_b0	40	960	423
	Concatenated feature vector	40	2240	1067

**Table 6:** Classification accuracies attained for each HAR datasets before and after applying BBA based feature subset selection.

Dataset	Model	Accuracy without wrapper(%)	Accuracy after wrapper(%)
HARTH	Mobilenet_v3_large	60	87
	Efficientnet_b0	68	86
	Concatenated feature vector	68	88.89
KU-HAR	Mobilenet_v3_large	73	97
	Efficientnet_b0	78	96
	Concatenated feature vector	77	97.97
HuGaDB	Mobilenet_v3_large	88	93
	Efficientnet_b0	88	92
	Concatenated feature vector	88	93.82

**Table 7:** Recall, Precision and F1-score values achieved after using BBA-based feature selection method for three HAR datasets.

Dataset	Model	Recall after wrapper	Precision after wrapper	F1-score after wrapper
HARTH	Mobilenet_v3_large	0.87	0.89	0.87
	Efficientnet_b0	0.86	0.89	0.85
	Concatenated feature vector	0.89	0.90	0.89
KU-HAR	Mobilenet_v3_large	0.97	0.97	0.97
	Efficientnet_b0	0.96	0.97	0.97
	Concatenated feature vector	0.96	0.97	0.96
HuGaDB	Mobilenet_v3_large	0.93	0.93	0.93
	Efficientnet_b0	0.92	0.92	0.92
	Concatenated feature vector	0.93	0.94	0.93

### Loss plot

**Figure 12:**
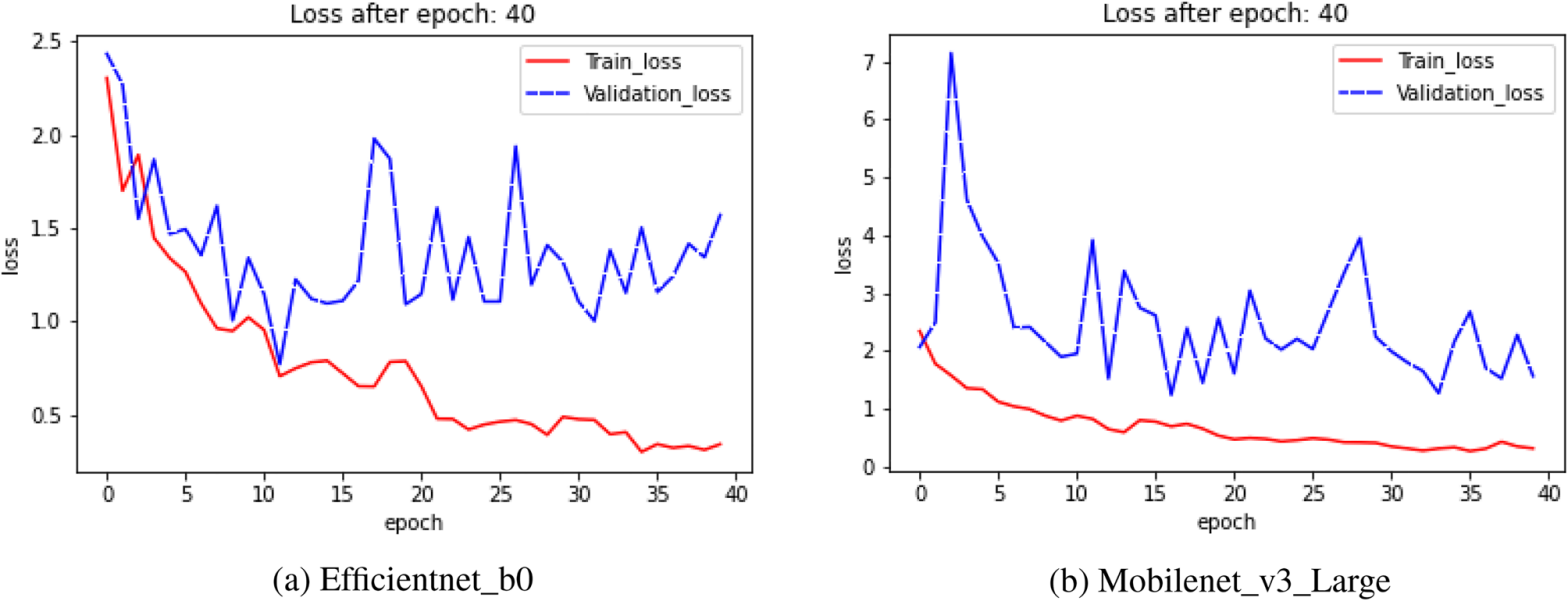
Graph showing the Loss plot generated by (**a**) Efficientnet_b0 and (**b**) Mobilenet_v3_Large transfer learning models on HARTH dataset.

**Figure 13:**
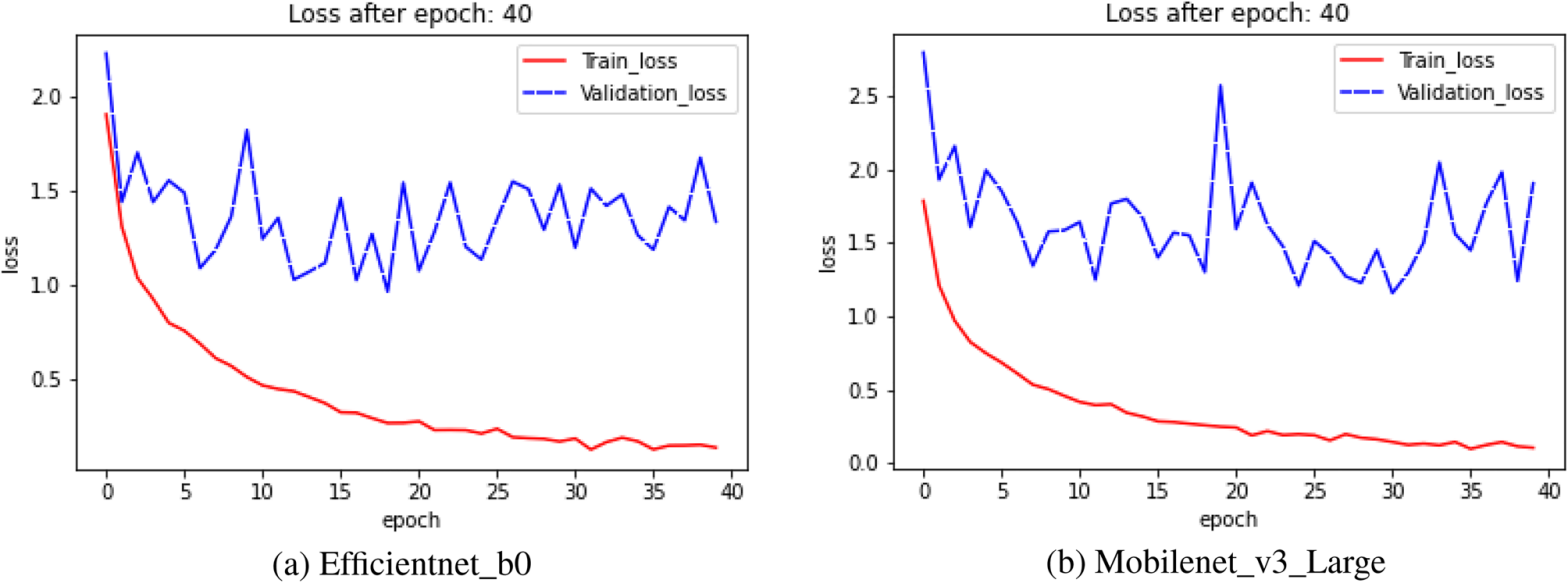
Graph showing the Loss plot generated by (**a**) Efficientnet_b0 and (**b**) Mobilenet_v3_Large transfer learning models on KU-HAR dataset.

**Figure 14:**
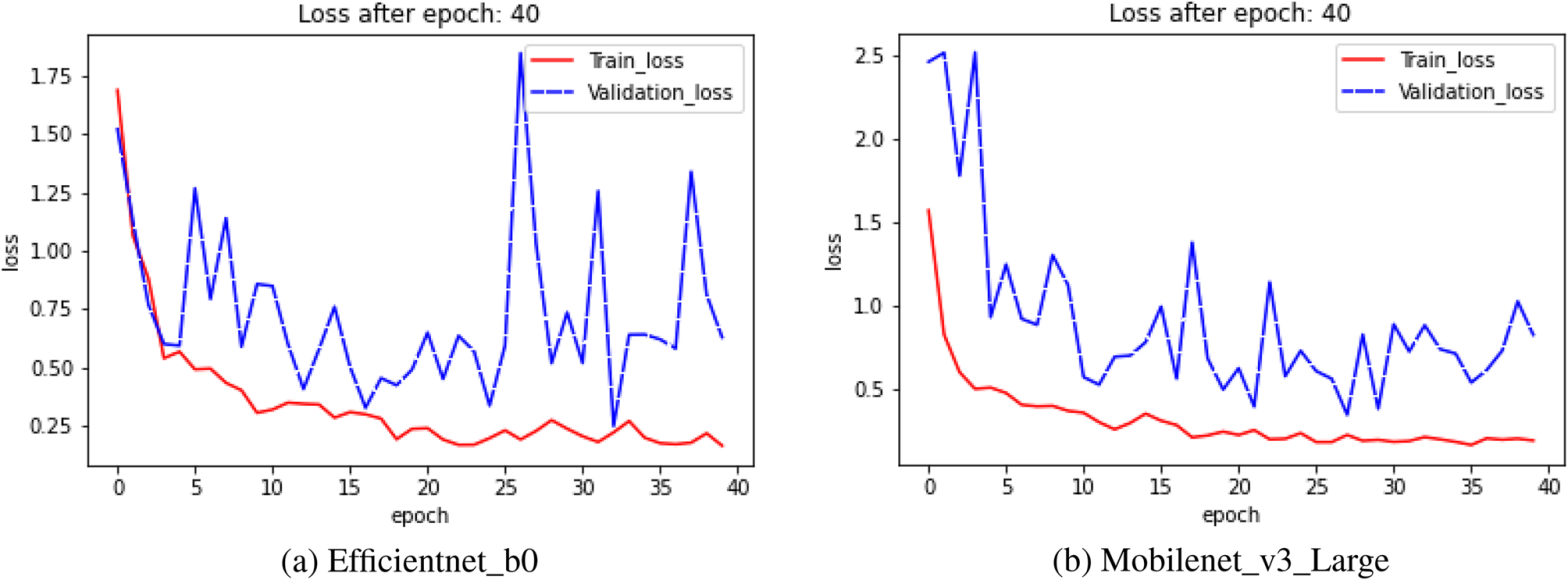
Graph showing the Loss plot generated by: (**a**) Efficientnet_b0 and (**b**) Mobilenet_v3_Large transfer learning models on HuGaDB dataset.

The loss plot gives us a summary of the training process and the network’s learning process. The loss function is used to determine the quantitative loss measure at the designated epoch across all data items throughout the course of an epoch. Figures 12, 13, and 14 show the train and validation loss plots of the combination of each model performed on the HARTH, KU-HAR, and HuGaDB datasets respectively. The models are trained using each HAR datasets for 40 epochs, as is already indicated. Figures 12, 13, and 14 show that the loss values decreases with respect to epoch, indicating that the models are picking up on the input item and increasing the prediction probability. Figures 12 and 13 show that the training loss curve converges after 35 epochs for both HARTH, KU-HAR datasets, whereas Figure 14 shows that the training loss curve for the Efficientnet_b0 model converges after 35 epochs whereas in case of Mobilenet_v3 model, the loss converges after 20 epochs.

### Analysis of confusion matrix

**Figure 15:**
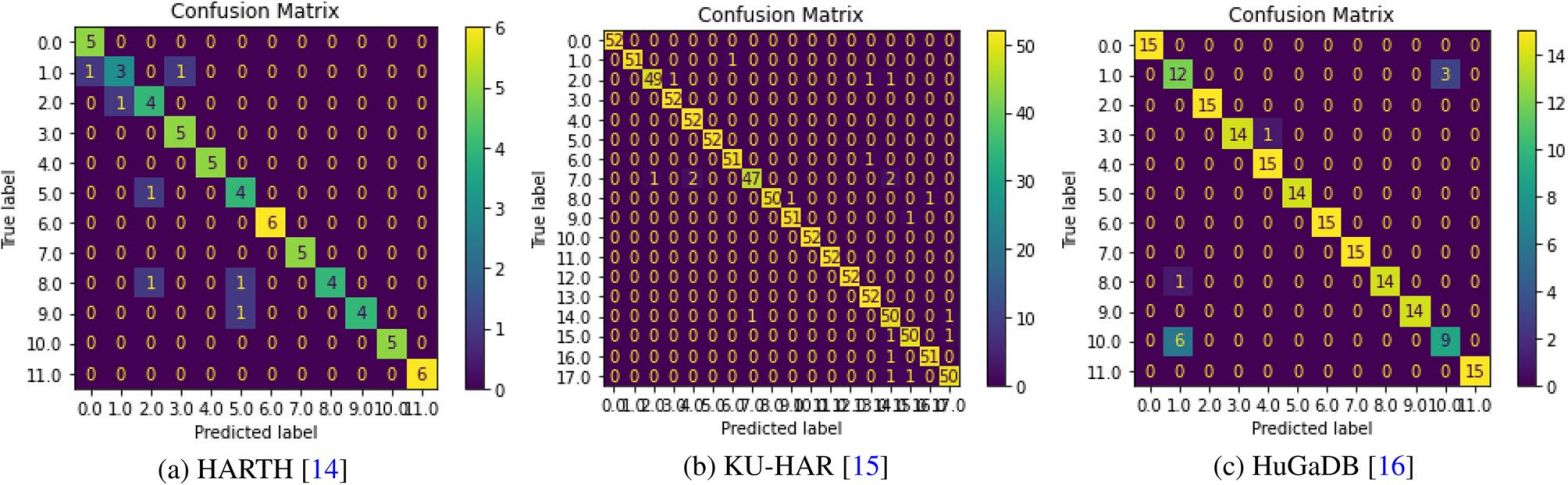
Graph showing the confusion matrices generated by our proposed HAR framework on: (**a**) HARTH, (**b**) KU-HAR and (**c**) HuGaDB datasets.

A snippet of the classification problem’s prediction outcomes is called a confusion matrix. Each activity class’s share of successful and failed predictions is indicated with count values. Figure 15 illustrates how the magnitude of the confusion relies on the number of activity classes contained in the HAR dataset. The confusion matrix for HARTH and HuGaDB datasets is 12 by 12, whereas the confusion matrix for KU-HAR dataset is 18 by 18. Each row in the confusion matrix stands for an anticipated activity class. Every column in the confusion matrix corresponds to a unique class. The diagonal numbers indicate how frequently the samples are successfully categorised. The samples that the model is unable to correctly categorise are the numbers that are not on the diagonal.

According to the confusion matrix (Fig. 15a) for the HARTH dataset, it can be seen that two instances of ‘Running’ activity class are mistaken for ‘Walking’ and ‘Climbing stairs’ classes, which are both continuous activities that involve a person moving from one place to another. Other activity classes, on the other hand, are correctly classified. Out of 52 samples in the KU-HAR dataset, the confusion matrix (shown in Fig. 15b) illustrates that five times the ‘Pick’ activity class which contain vertical acceleration, are incorrectly classified as ‘Talk-Sit’, ‘Stand-Sit’, and ‘Run’ activity classes. The other activity classes in the KU-HAR dataset are all accurately categorised. For the confusion matrix (shown in Fig. 15c) for the HuGaDB dataset, it can be examined that due to the linear acceleration of both activities, out of 15 samples, the ‘Down-By-Elevator’ activity class is incorrectly identified as ‘Running’ activity class for 6 times out of 15 samples whereas the ‘Running’ activity class is incorrectly classified as ‘Down-By-Elevator’ class in three times out of 15 times.

### BBA parameter setting

BBA used the concatenated feature vector from both the transfer learning models to choose the best feature subset. Execution parameters for BBA are shown below:

- Number of agents: 30
- Number of iterations: 100
- Validation data percentage: 20%

**Figure 16:**
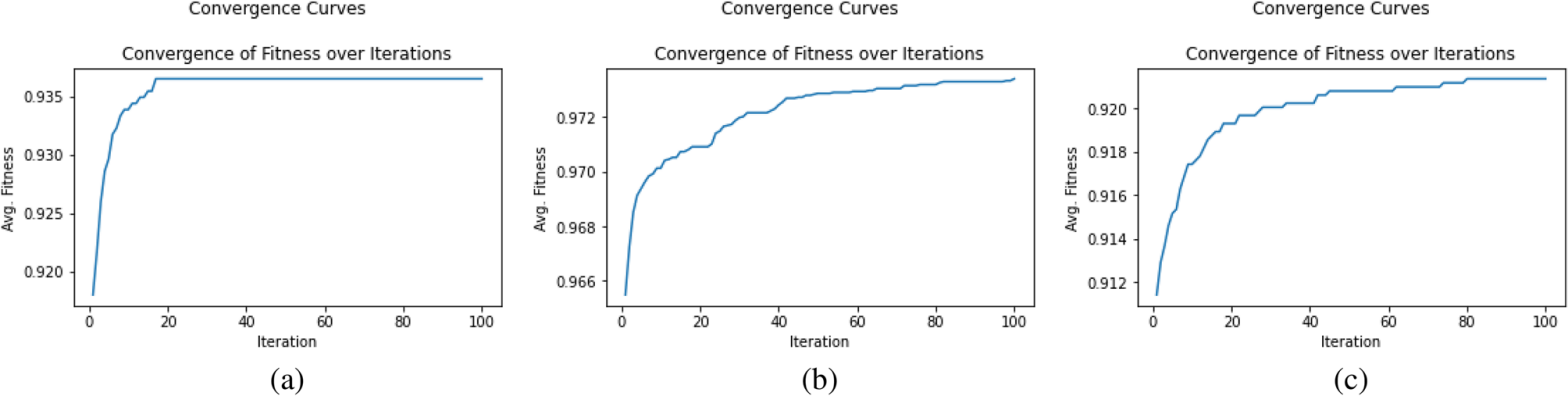
Convergence curves to show average fitness over iterations after applying BBA on concatenated feature set for: (**a**) HARTH dataset, (**b**) KU-HAR dataset and (**c**) HuGaDB dataset.

Figure 16 illustrates how the average model fitness rose for each iteration. Figure 16a shows the convergence curve for the HARTH dataset whereas Figs. 16b and c show the convergence curves for the KU-HAR and HuGaDB datasets respectively. It can be examined from Figure 16a that the convergence curve becomes flat after 20 iterations whereas in case of the KU-HAR and HuGaDB datasets, the convergence curve becomes flat after 80 iterations.

### Comparison with existing models

Table 8 compares the performance of our wrapper-based deep feature optimization HAR model with few earlier HAR models proposed till date for the three HAR datasets. As shown in Table 8, our proposed wrapper-based deep feature optimization HAR model obtains classification accuracies of 88.89%, 97.86%, and 93.26% for HARTH, KU-HAR, and HuGaDB datasets respectively. The results indicated that the wrapper-based deep feature optimization model, proposed for HAR problem, performs significantly better than all the earlier efforts on HAR.

**Table 8:** Performance comparison (in terms of classification accuracies) of our proposed wrapper-based deep feature optimization method with some existing HAR works implemented on the HARTH, KU-HAR, and HuGaDB datasets.

Dataset	Researcher	Accuracy(%)
HARTH	Logacjov et al.^14^	81
	**Proposed wrapper-based deep feature optimization HAR framework**	**88.89**
KU-HAR	Sikder and Nahid^15^	89.67
	**Proposed wrapper-based deep feature optimization HAR framework**	**97.86**
HuGaDB	Gochoo et al.^36^	92.5
	Logacjov et al.^14^	81
	Filtjens et al.^41^	83.8
	Javeed et al.^37^	92.5
	Fang et al.^38^	79.25
	Sun et al.^39^	88
	Kumari et al.^40^	91.1
	**Proposed wrapper-based deep feature optimization HAR framework**	**93.82**

Bold indicates the least number of features selected and highest values of accuracy, Recall, Precision and F1-score attained for each of the HAR datasets.

### Comparison with other optimization algorithms

Utilizing five well-known metaheuristic optimization algorithms, including the Cuckoo Search Algorithm (CSA)^50^, Equilibrium Optimizer (EO)^51^, Genetic Algorithm (GA)^52^, Gravitational Search Algorithm (GSA)^53^, and Grey Wolf Optimizer (GWO)^54^, the proposed BBA-based deep feature optimization model has been tested in the current work. Table 9 enlists the performance results achieved by applying all the above meta-heuristic optimization algorithms. It can be noticed from Table 9 that for both the HuGaDB and KU-HAR datasets, the proposed wrapper-based BBA has given the best classification accuracy with the least number of selected optimal features. However, in case of HARTH dataset, the CSA selects only 1 feature less than as compared to the proposed BBA but despite that our proposed model produces an accuracy gain of approximately 2%. This justifies the trade-off between classification accuracy and the number of optimal features selected which is negligible compared to the former. Following a comparison of the number of selected optimal features with the corresponding classification accuracies, it can be said that the BBA is one of the best-suited wrapper algorithm for each of the three HAR datasets.

**Table 9:** Comparison of our proposed BBA based feature selection methodology with various well-known meta-heuristic feature selection algorithms used in the literature. (Here, bold indicates the least number of features selected and highest values of accuracy, Recall, Precision and F1-score attained for each of the HAR datasets).

Dataset	Optimization algorithm Used	Number of features selected	Accuracy (%)	Recall	Precision	F1-Score
HARTH	Cuckoo Search Algorithm(CSA)^50^	**905**	87.3	0.873	0.889	0.873
	Equilibrium Optimize(EO)^51^	1105	88.29	0.883	0.891	0.888
	Genetic Algorithm(GA)^52^	1066	88.48	0.885	0.894	0.883
	Gravitational Search Algorithm (GSA)^53^	1135	87.48	0.875	0.894	0.883
	Grey Wolf Optimizer(GWO)^54^	1346	88.49	0.885	0.891	0.888
	**Proposed Model (BBA)**	906	**88.89**	**0.889**	**0.901**	**0.888**
KU-HAR	Cuckoo Search Algorithm(CSA)^50^	1208	97.54	0.975	0.956	0.955
	Equilibrium Optimize(EO)^51^	1125	97.76	0.955	0.958	0.958
	Genetic Algorithm(GA)^52^	1157	97.65	0.956	0.957	0.958
	Gravitational Search Algorithm (GSA)^53^	1106	97.86	0.959	0.959	0.959
	Grey Wolf Optimizer(GWO)^54^	1470	97.76	0.958	0.958	0.957
	**Proposed Model (BBA)**	**1049**	**97.97**	**0.965**	**0.965**	**0.965**
HuGaDB	Cuckoo Search Algorithm(CSA)^50^	1113	91.57	0.916	0.922	0.916
	Equilibrium Optimize(EO)^51^	1100	91.01	0.910	0.916	0.911
	Genetic Algorithm(GA)^52^	1047	91.57	0.916	0.922	0.916
	Gravitational Search Algorithm(GSA)^53^	1122	91.01	0.910	0.916	0.911
	Grey Wolf Optimizer(GWO)^54^	1423	91.01	0.910	0.915	0.912
	**Proposed Model (BBA)**	**1017**	**93.82**	**0.939**	**0.943**	**0.939**

### Statistical significance test: McNemar’s test

In the preceding section, we thoroughly examined the performance of our proposed model on three HAR datasets and found that the proposed framework of the two base models outperforms each of them in terms of accuracy. To specifically show the effectiveness of our recommended Wrapper based classification model over the basic models, we ran a statistical significance test^55^ known as the McNemar’s test^56^.

The results of McNemar’s test performed on the three HAR datasets-HARTH, KU-HAR, and HuGaDB datasets are presented in Table 10. McNemar’s test’s *p*-value should ideally be below 5% in order to reject the null hypothesis^57^, and Table 10 clearly shows that this is the case in every scenario where the *p* value is less than 0.05. Therefore, in every instance, the null hypothesis is rejected. As may be deduced from the aforementioned statistical tests, the findings obtained by the base models and suggested ensemble model are statistically significant, i.e., not equal. This explains why the proposed HAR framework integrates the supplementary details supplied by the individual classifiers and produces better predictions, setting the overall wrapper-based feature optimization model apart from all of the individual transfer learning models.

**Table 10:** Results of the McNemar’s test conducted on MobileNet_v3_large and Efficientnet_b0 transfer learning models: In every situation, the null hypothesis is rejected.

McNemar’s Test	*p*-value		
Model compared with	HARTH^14^	KU-HAR^15^	HuGaDB^16^
MobileNet_v3_large^43^	0.0395	0.0287	0.0368
Efficientnet_b0^33^	0.0426	0.0343	0.0156

## Conclusions & future works

HAR is still one of the challenging research areas for the research community due to the dynamic nature of the data since there is no predefined number of human activities list. Three distinct HAR datasets, namely, HARTH containing 12 human activities, KU-HAR containing 18 human activities, and HuGaDB containing 12 human activities, have been utilized in this study. It is to be noted that all the three HAR datasets, used in the present work, have a different collection of human activities. Developing distinct techniques for each HAR datasets is not feasible. Therefore, the goal is to create a generic HAR model that applies to any acceleration and gyroscope based sensor data. Here, a combination of two distinct kinds of transfer learning models along with a wrapper-based feature selection approach are used to achieve the same goal. The three recently developed benchmark HAR datasets namely, HARTH, KU-HAR, and HuGaDB, are used to test the proposed model, and the classification accuracies are found to be 88.89%, 97.9%, and 93.82%, respectively. The experimental results performed better than most recent state-of-the-art findings. It is to be noted that almost 52%, 45% and 60% of original number of deep features for HuGaDB, KU-HAR and HARTH datasets respectively, have been selected by the present wrapper-based feature selection method using BBA. On the other hand, a total of about 21%, 20% and 6% improvement in the overall classification accuracies have been attained by the proposed wrapper-based deep feature optimization methodology on HuGaDB, KU-HAR and HARTH datasets respectively. This is one of the major advantages of our proposed HAR framework.

The proposed model can be tuned in future by applying the following approaches:

- Filter method can be used to rank the features and instead of sending all features to the wrapper, they can be sent ranked-wise.
- The proposed model can be integrated with different type IoT and smartphone devices for real-time HAR prediction.
- The proposed HAR model can be validated on other publicly available datasets available in literature.

The primary contribution of this work is the proposed two-fold architecture model, which outperforms the prior state-of-the-art HAR models proposed in the literature. It offers a strong basis for combining a transfer learning deep feature extraction model with a wrapper-based feature selection approach that has already been trained for HAR. In the coming future, it may be integrated with many IoT and smartphone platforms, which is advantageous in a variety of fields like mobile app development, healthcare, and many more.

## Data Availability

No datasets are generated during the current study. The datasets analyzed during this work are made publicly available in this published article.

## Code availability

The source codes related to the current work are made publicly available at: https://github.com/ghoshraghu2610/HAR_WITH_TRANSFER_AND_WRAPPER.git.
